# Deep hypothermic preservation of autologous skin in the treatment of large-area circumferential multi-plane degloving trauma: a pilot study of 2 cases

**DOI:** 10.1007/s10561-018-09745-4

**Published:** 2019-01-12

**Authors:** Lijie Tian, Xianglu Ji, Ting Chen, Feng Qi, Furong Tian, Qiang Yao, Feng Tian

**Affiliations:** 1 Department of Hand and Foot Surgery, Shengjing Hospital of China Medical University, Shenyang, 110004 People’s Republic of China; 2 Department of Hand and Foot Surgery, Shenwei Hospital Shenyang, Shenyang, 110000 People’s Republic of China; 3 Department of Hand Surgery, Central Hospital of Shenyang Medical College, Shenyang, 110024 People’s Republic of China

**Keywords:** Deep hypothermic preservation, Autologous skin, Large-area degloving trauma

## Abstract

To evaluate the clinical outcome of deep hypothermic preservation of autologous skin in the treatment of large-area skin avulsion. Medium or full thickness-skin slices were harvested from large avulsion flaps between July and November 2017. They were stored in liquid nitrogen by vitrification. After the patient’s condition became stable and the growth of the wound granulation tissue was satisfactory, the frozen skin slices were reheated quickly and replanted to the wound. Autologous skin that had been kept by deep cryopreservation had a high survival rate when grafted. It did not create new trauma or bring additional pain to patients. Yet it could shorten the course of treatment and reduce the medical cost for patients. It is an effective and economical way to treat large-area skin avulsion.

## Background

The recent decade witnessed a dramatic rise in the number of severe mechanical and traffic accidents. The accidents sometimes led to large-area skin degloving injuries, which were often complicated by deep tissue damages, fractures, crucial organ injuries, hemorrhagic and traumatic shock.

At present, the commonly-used clinical treatments include immediate skin grafting in situ and suture in situ. The first choice routinely necessitates long-time surgeries, which imposes tremendous risk to patients suffering severe shock or important organ damages. The second will inevitably cause partial or subtotal necrosis. Besides, since new skin graft needs to be harvested from other sites of the body, the patient will have to sustain new trauma. The donor sites are left with a large scar, apart from suffering intense itching and other sequelae.

In 2012, in cooperation with the National Skin Bank of Italy, Italian doctor Mario Dini succeeded in treating large-area skin degloving trauma by vitrification (Dini et al. 2012). The preserved skin, previously taken from the patient, was replanted to his lower extremities after the stabilization of his condition. The success of Dr. Dini shed new light upon the skin grafting strategy in patients with unstable condition.

In this article, we report two successful cases of treating large-area degloving trauma through cryopreservation of autologous skin grafts in liquid nitrogen. In both cases, the preservation was exclusively done by our own staff instead of a third-party institution.

## Clinical materials

### Case 1

**Fig. 1:**
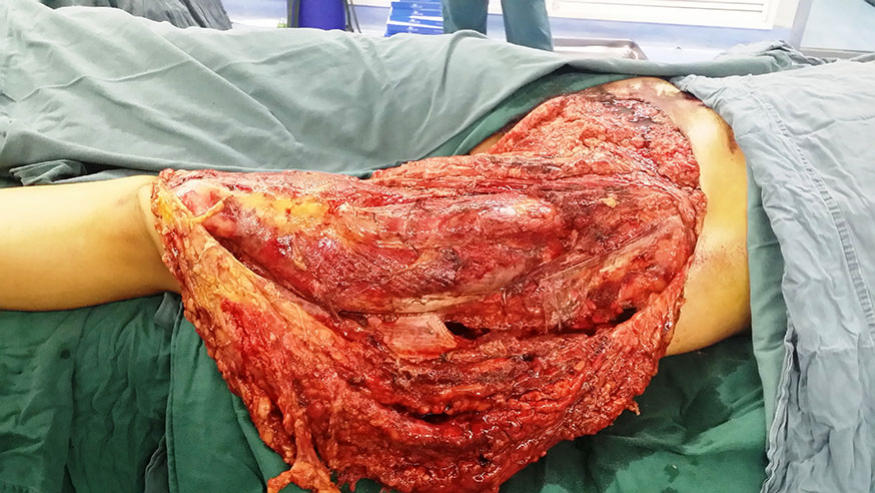
Case 1. The avulsion of the skin was from the lower left abdomen to the knee joint, and covers an area of about 2200 cm^2^. Circumferential multi-plane injury was observed, accompanied by large amount of muscle and fatty fascia damage

A 28-year-old female was injured by forklift crush that caused skin degloving in the lower left abdomen, as well as the entire thigh and the knee joint. The total damaged area was about 2200 cm^2^. The wound was contaminated and accompanied by circumferential multi-plane injury. The patient was also suffering traumatic and hemorrhagic shock (Fig. 1), with the blood pressure: 76/39 mmgh, p:112 beats/min, HGB:62 g/L, albumin: 23.5 g/L, total protein: 46 g/L, HCT:24.3%, PT:15.3 s. Emergent operation was performed. After wound debridement and hemostasis, the scale of the skin ischemia was decided by puncturing and trimming the skin edge: the fringe of the ischemia lies where there’s no bleeding (Ziv et al. 1990). Skin flap lack of blood supply was excised. The remaining skin was pulled together by suture to reduce the wound, and the remaining wound was covered by the Vacuum Sealing Drainage (VSD, Waystech, Guangzhou, China).

Medium-thickness skin grafts were harvested from the excised flap with a rolling knife. The skin grafts were rolled up between two layers of gauze. The gauze was then stitched up with a needle and steel wire to form a cylinder. At one end of the cylinder, an appropriate length of the steel wire was reserved, so that the cylinder could be readily pulled out of the liquid nitrogen tank once necessary. The cylinder was first immersed into the antifreeze solution [composed of 20% dimethyl sulfoxide, 6% glycol propylene and Kreb Ringer solution (Zhu et al. 1991)] for 30 min. Then it was immediately transferred into the liquid nitrogen tank for storage.

**Fig. 2:**
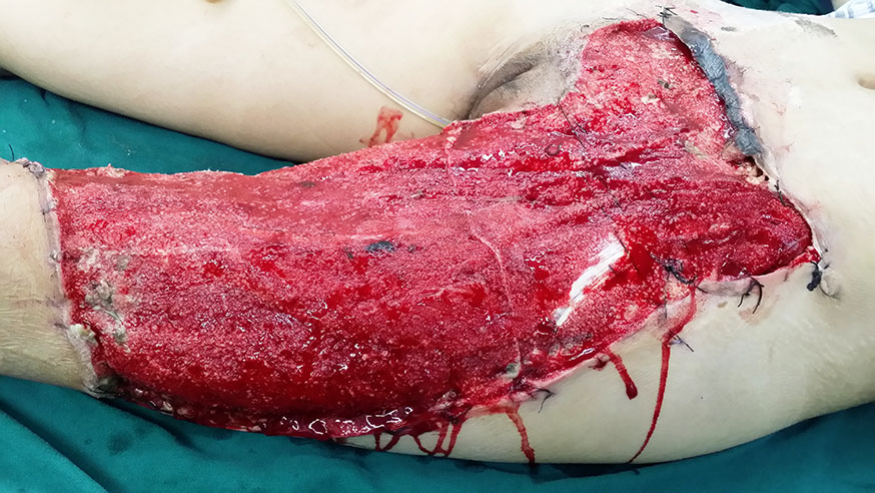
Case 1. Granulation tissue grew fresh and well, 13 days after the injury and the application of VSD

During and after the operation, blood transfusion and albumin infusion were carried out. Antibiotics were also used to prevent infection. Thirteen days after the injury, the hemoglobin and albumin of the patient returned to normal. PT:14.1 s, and the patient had no fever. After the removal of VSD, fresh granulation tissue could be seen. No infection or active bleeding was observed (Fig. 2).

The second operation was carried out. The preserved skin grafts were taken out of liquid nitrogen. They were rapidly thawed in 42–45 °C normal saline for 1 min. The grafts were washed for 3 times, 5 min each time with saline, then immersed in normal saline of the room temperature for 15 min. The preserved skin grafts showed minimal change as to color, wholeness and softness. Skin grafts that had sustained the least damages were chosen for the operation. They were transplanted to the wound, and then covered with VSD. After the operation, the skin grafts preserved were left about 150 cm^2^. The entire surgery lasted for 5.5 h.

**Fig. 3:**
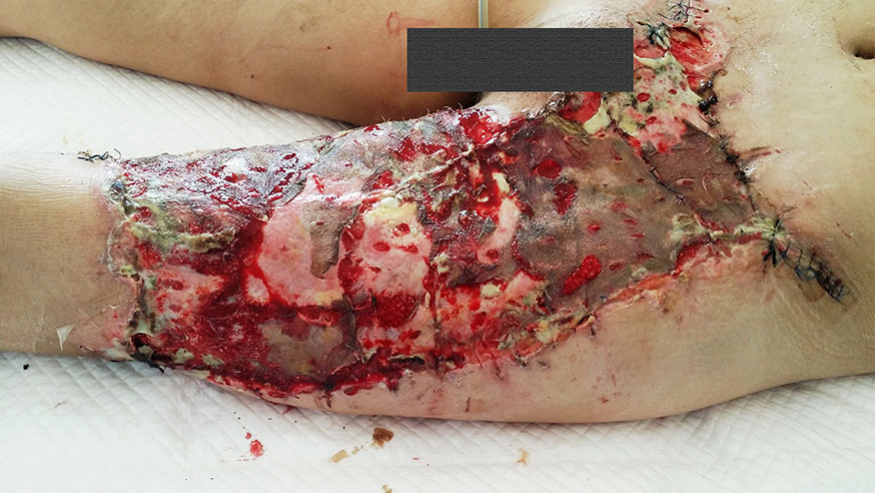
Case 1. Nine days after the autologous skin graft, most grafted skin survived, with necrosis happening only on a very small scale (yellow parts). (Color figure online)

**Fig. 4:**
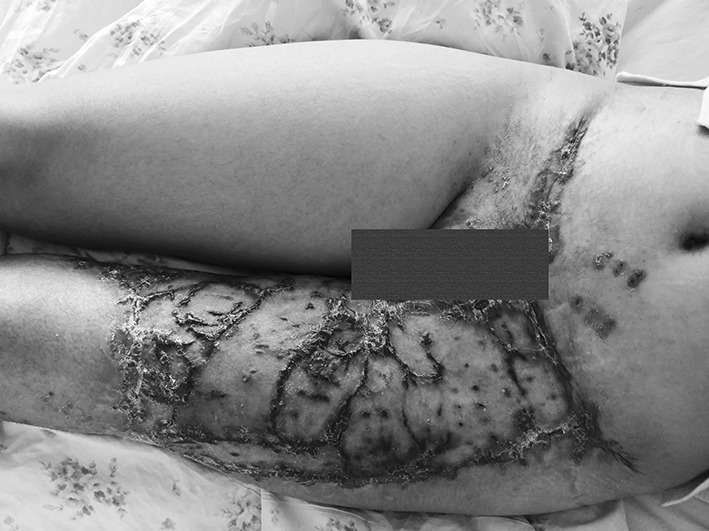
Case 1. 40 days after skin grafting, the wound was healed, and the skin survived well without infection and vesicles

**Fig. 5:**
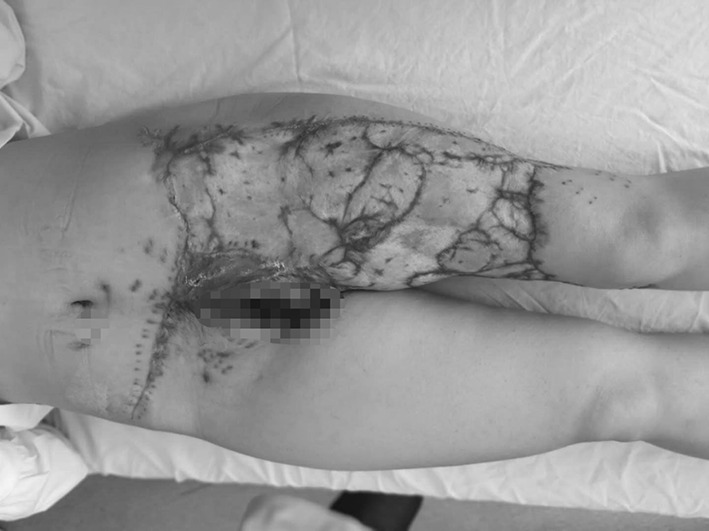
Case 1. One year later, the skin color changed from flushing to normal color

**Fig. 6:**
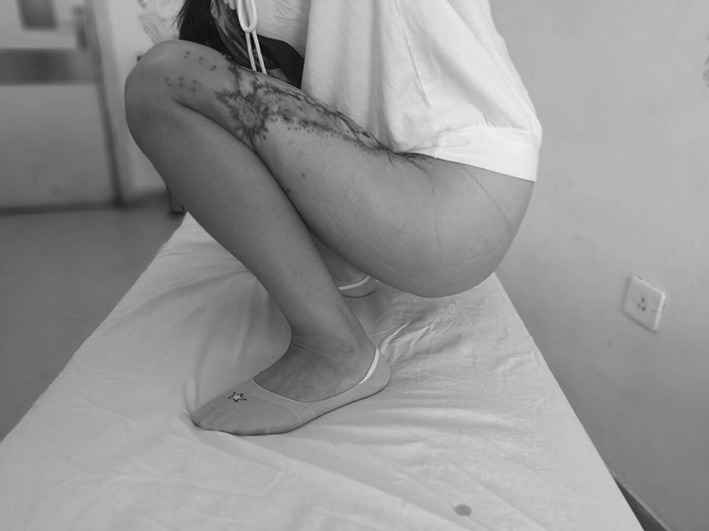
Case 1. One year later, the hip was slightly restricted

Nine days after the second operation, the VSD was removed. Spotted necrosis was found on the edge of the skin graft. But overall, the survival rate of the skin graft had reached 95%. The survived part showed no difference from the surviving skin in a grafting operation that uses healthy skin tissue (Fig. 3). For the next 40 days, the dressing was changed regularly and the wound was kept dry until the healing was complete (Fig. 4). One year later, the skin color changed from flushing to normal color and the hip flexion was slightly restricted (Figs. 5, 6).

### Case 2

**Fig. 7:**
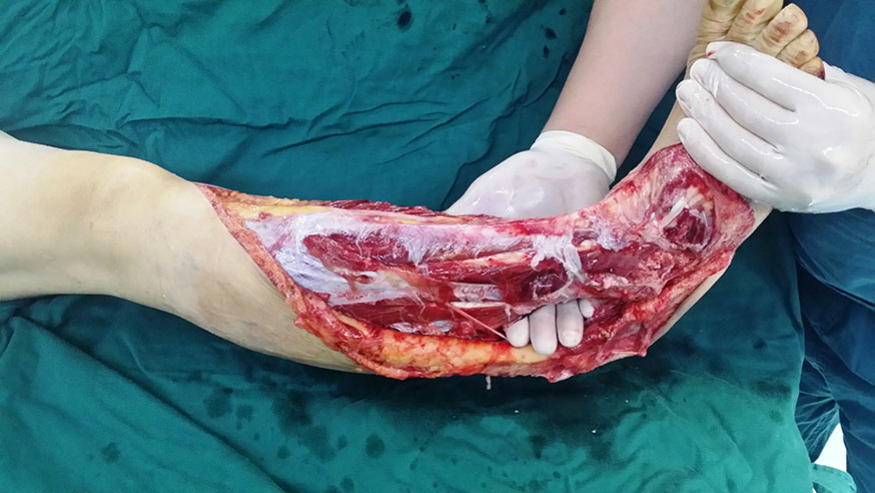
Case 2. The skin was avulsed from below the left knee to the middle part of the foot including the medial and lateral malleolus. Circumferential multiplane degloving took place, with a large amount of muscle and fat fascia damage

A 56-year-old female was run down by a car. Her right leg suffered extensive degloving injury from the knee right down to the back of the foot. The total wounded area, which added up to 1200 cm^2^, was severely contaminated and accompanied by circumferential multi-plane injury, fibula exposure and partial muscle rupture (Fig. 7). Blood pressure: 109/61 mmHg, heart rate: 84 beats/min, HGB: 98 g/L, HCT: 30.4%, albumin: 25.8 g/L, total protein: 52 g/L, PT: 12.2 s.

We did roughly the same procedures as with case 1. The only difference was that the skin was harvested with a pair of scissors in the form of full-thickness skin graft. There was no break in the entire graft.

Ten days after the injury, we found that the granulation in the wounded area had been growing relatively slowly and that there were a small amount of residual necrotic tissues. Debridement was carried out, as well as a change of the VSD coverage.

**Fig. 8:**
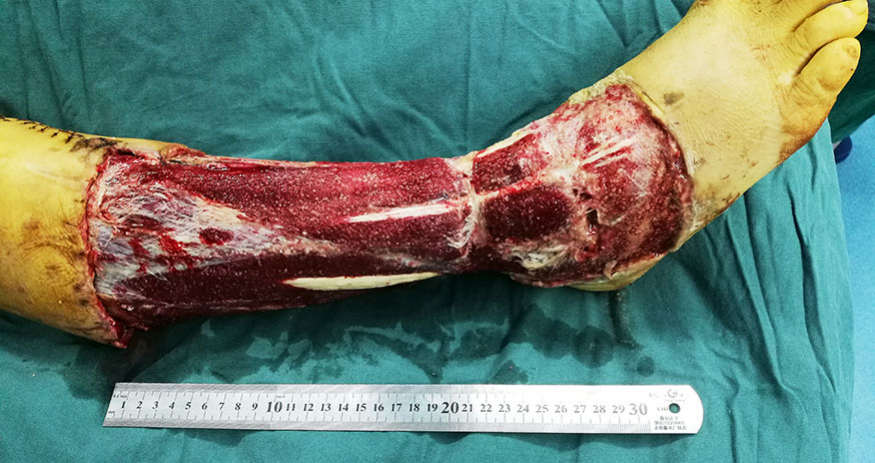
Case 2. Sixteen days after the injury, and after twice debridement and application of VSD (the first time, during the emergency operation on the day of the injury; the second time, 10 days after the injury) the granulation tissue grew well, with a small amount of tendon was exposed

Skin grafting was carried out 16 days after the injury. By that time, fresh granulation tissue had fully covered the wounded area apart from the medial malleolus. There was no infection or active bleeding (Fig. 8).

The skin graft preserved in liquid nitrogen was rewarmed using the same method as in case 1. The graft was then replanted to the wounded surface, with the edge of the skin pruned and the entire grafted area covered with VSD. The remaining unused part of the skin graft was fixed with 4% polyformaldehyde, for pathological examination.

**Fig. 9:**
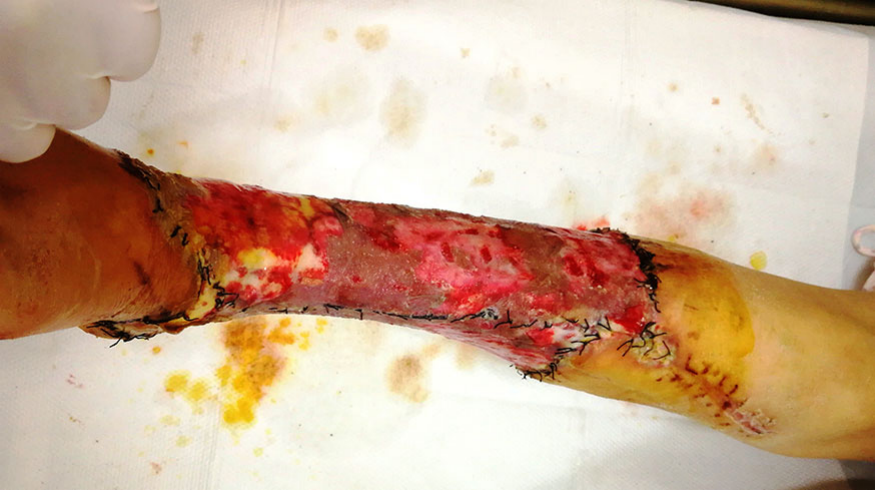
Case 2. Ten days after autologous skin grafting, the VSD was removed. Most of the skin survived, with necrosis on a small scale (the yellow area). (Color figure online)

The VSD was removed 10 days after the operation. A small area of the anterior of the ankle failed to take hold, while about 96% of the skin graft proved viable (Fig. 9).

**Fig. 10:**
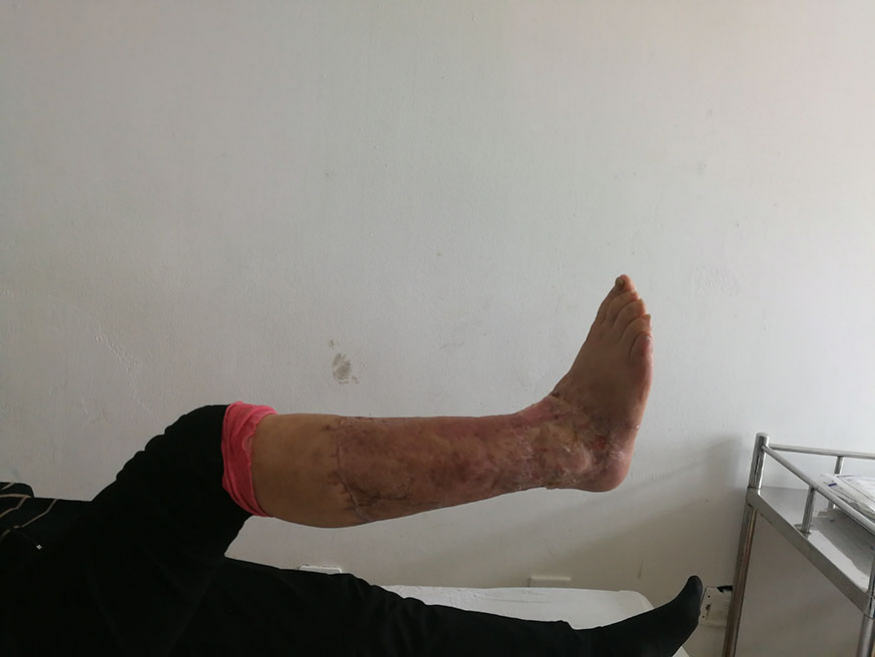
Case 2. After 1 years of follow-up, the skin texture and the function was acceptable

After 1 years of follow-up, the skin texture and the function was acceptable (Fig. 10).

**Fig. 11:**
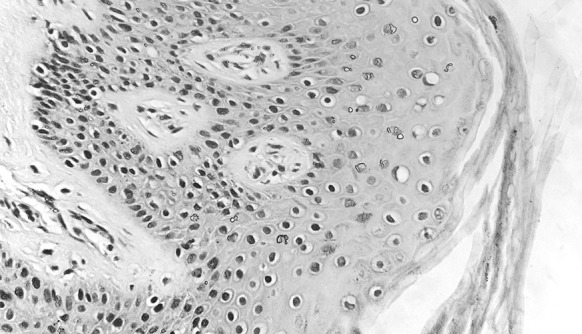
Case 2. Pathology (HE staining, 400 ×) performed on skin previously preserved for graft and ultimately left unused. The structure of the tissue and cells was integrated and the nucleuses were slightly enlarged

Since one large skin grafts instead of several small skin grafts was preserved and then grafted, there was less scar and the final appearance of the operated area looked remarkably better than it was with case 1. The wound was kept dry and the regular change of dressing was maintained until the wound completely healed 28 days after the third operation. Pathology performed on the remaining skin graft showed that the cells and the tissue structure were complete, but the size of the nucleus increased slightly (Fig. 11).

After a 1-year follow-up, no systemic or local complications such as hepatorenal toxicity, tumors, dermatitis and so on were found in the both of the two patients.

## Discussion

### Classification

In a report published in 2009, Z.M. Arnez categorized skin avulsion into 4 types: (1) abrasion/avulsion; (2) non-circumferential degloving; (3) circumferential single-plane degloving; (4) circumferential multi-plane degloving. Type 4 was the most serious of all, which often came to unsatisfactory results from the initial operation. Therefore, a multi-staged reconstruction process was often required for such cases (Arnez et al. 2010).

### Pathology of large area of skin degloving trauma

The damage was very complex. Large areas of skin and subcutaneous tissue were avulsed from the deep fascia and above. While deep tissue such as muscles and tendons might remain intact, they could also be contused and become exposed to varying degrees. In severe cases, fracture as well as bone or joint exposure could also happen.

As the result of various forces the skin sustained during an injury, including crushing, pulling and tearing, the skin itself suffered damages and avulsions. The blood vessels underneath that nourished the skin were also subject to extensive bruises and breakage.

The blood supply for skin of the extremities comes from direct cutaneous artery or musculocutaneous artery, both of which originate from the deep artery stem before winding through the deep fascia to reach the reticular layer of the superficial fascia. The degloving trauma severely destroys the direct cutaneous artery or musculocutaneous artery.

Sometimes, there is still a wide pedicle connected to the normal tissue, so the blood supply is still available. However, as time goes, both thrombosis and necrosis have a high incidence. Therefore, to suture the avascular flap in situ is potentially dangerous (Xu et al. 2006).

### The scarcity of the operation time

Large-area skin-degloving traumas are often accompanied by craniocerebral, thoracic and abdominal injuries, which may lead to traumatic and hemorrhagic shock. To save the lives of patients and to shorten the operation time, priority should be given to the emergent surgery of important organs. Meanwhile, the surgeon may remove the subcutaneous hematoma and necrotic fat of the avulsed skin flap before suturing the flap in situ and applying the bandage with pressure. Measures should be taken against possible necrosis and late-presenting infections (MacCollum 1938; Hudson et al. 1992).

Due to the destruction of the blood supply, ischemic necrosis would happen and the avulsed skin flap would take on the color and texture of black leather a week later. In such cases, surgeons need to remove the necrotic skin, hematocele, liquefaction and infected tissue under the skin flap, and wait for fresh granulation tissue to grow. Once fresh granulation tissue covers the wound surface, the wound can readily be closed up by transplanting the skin grafts harvested from the healthy part of the body.

Apart from lengthening the course of treatment, the multiple operations would also add to the trauma and sufferance of the patients.

### Disadvantage of immediate backgrafting

Large-area skin-degloving traumas are often caused by mechanical grinding and crushing, which results in not only the skin damage, but also in injuries to the superficial fascia, deep fascia, muscle, nerve, vessels, bones and joints. In such cases, debridement is relatively difficult especially when there is tissue necrosis and/or severe contamination. Sometimes two to three debridements are required to obtain a wound that can undergo skin grafting. Inadequate debridement of the wound is bound to reduce the survival rate of skin grafting (Minten et al. 1992; Khan et al. 2004; Meara et al. 1999).

For patients with shock or vital organ injuries, priority should be given to life-saving procedures. If medium- or full-thickness skin graft is to be harvested from the avulsion skin, it will greatly lengthen the operation time. Therefore, considering the wound condition and the operation time, it is inadvisable to replant the skin immediately.

Due to excessive bleeding and low perfusion state, it is hard to estimate the tissue vitality and the perfusion of the skin in case of an extensive avulsion. A thorough debridement is harder in this situation. Besides, the survival rate of the grafted skin will be reduced since the wound is large and associated with multi-plane soft tissue injury. Prolonged coagulation time renders bleeding hard to stop. After the primary skin grafting, there will still be residual avascular tissue, infection and subcutaneous hematoma. All these factors will contribute to the low survival rate of the grafted skin.

There is sufficient time to increase the patient’s hemoglobin and albumin level, restore electrolyte homostasis and improve the patient’s coagulation time, which in turn will improve the nutritional status of the wound. After the grafting operation, the bleeding stops spontaneously within a few days. Improved nutritional status and less exudation are also more conducive to the survival of the skin.

### Vitrification of human skin

The application of vitrification in the preservation of cells and tissues has been increasing since the successful preservation of mouse embryos reported by Fahy et al. (1984).Vitrification is the process of transforming a substance into a glass-like amorphous body (glass state), resulting in no crystal structure in the final form. When the cells are cooled by vitrification, the intra- and extracellular water molecules would not crystallize. Since the cell structure is not damaged, the cells can remain alive. This technique is often used in high-difficulty cryopreservation of eggs, ovarian tissue, human embryonic stem cells and the establishment of a skin bank.

At present, most of the skin from the skin banks is used for allograft. The aim of vitrification is to produce no ice crystals in the cooling process, as the temperature drop from room temperature to − 120 °C. Once the viable tissue is kept in a temperature below − 120 °C, the intracellular water stops to crystallize. In liquid nitrogen, where the temperature reaches − 196 °C, metabolism of the cells is effectively put to rest. In this case, the cell can survive much longer than it would have done in normal condition.

In 2012, an Italian doctor Mario Dini first reported the successful cryopreservation of autologous degloving skin and its subsequent replanting to the patient’s wound 21 days after the initial operation (Dini et al. 2012). However, there is no skin bank available in most hospitals. To solve this problem, we innovatively preserved the autologous skin in a liquid nitrogen tank for storage. The method is designed to improve the applicability of this technology.

The technology can effectively maintain the vitality of the skin, with the longest storage time for a certain skin graft ranging between 1 and 5 years (Fujita et al. 2000; Ben-Bassat et al. 2001). This time is enough for the improvement of the patient’s wound as well as his general condition. Therefore, the chances for necrosis will be dramatically reduced at the time of grafting. Since a second operation is often unnecessary, the patient is spared of further pain.

And since this procedure reduces the numbers of operation required, it helps to shorten the duration of hospitalization and therefore saves the cost. In addition, if the wound involves large exposed areas of bones, tendons and joints, skin flap graft is necessary. This technology allows for the use of cryopreserved autologous skin to cover the flap donor site, so there’s no need to harvest graft from healthy limbs.

### The effect of granulation tissue on the survival rate of skin grafting

The wound surface suitable for skin grafting should feature granulation that’s fresh and firm. It should also be fine and smooth, with a bright red color and prone to bleeding. Exudation should be minimal and there should be no edema.

The advantage of using VSD for temporary wound closure is that it can help suck the bleeding and exudates in time, therefore keeping bacteria growth in check. With VSD, frequent dressing change is not needed, so the patient suffers less. Generally speaking, 10–14 days would be enough for the granulation tissue to be ready for the grafting.

### Cost

The drugs and equipment used for the operation are relatively cheap. And the freezing and thawing process is easily applicable, and can be accomplished by surgeons themselves in the operating room. This also helps to save time needed for the transportation of the skin between the hospital and the skin bank, therefore reducing the skin ischemia time.

In summary, by presenting two successful cases, we advocated the cryopreservation of autologous skin grafts in the treatment of large-area degloving trauma, especially for the patients in unstable general condition. When facilitating the life-saving procedures, this protocol eliminated the necessity of skin harvest from other parts of the body and increased the survival rate of the graft. This time- and cost- efficient protocol may serve as a good choice for plastic surgeons.

## Abbreviations

VSD: Vacuum sealing drainage (Waystech, Guangzhou, China)
